# Reinforcement of Epoxidized Natural Rubber with High Antimicrobial Resistance Using Water Hyacinth Fibers and Chlorhexidine Gluconate

**DOI:** 10.3390/polym16213089

**Published:** 2024-10-31

**Authors:** Thidarat Kanthiya, Pornchai Rachtanapun, Siwarote Boonrasri, Thorsak Kittikorn, Thanongsak Chaiyaso, Patnarin Worajittiphon, Nuttapol Tanadchangsaeng, Sarinthip Thanakkasaranee, Noppol Leksawasdi, Yuthana Phimolsiripol, Warintorn Ruksiriwanich, Kittisak Jantanasakulwong

**Affiliations:** 1 Faculty of Agro-Industry, Chiang Mai University, Chiang Mai 50100, Thailand; thidaratkanthiya05@gmail.com (T.K.); pornchai.r@cmu.ac.th (P.R.); thachaiyaso.c@cmu.ac.th (T.C.); sarinthip.t@cmu.ac.th (S.T.); noppol@hotmail.com (N.L.); yuthana.p@cmu.ac.th (Y.P.); 2 Center of Excellence in Agro Bio-Circular-Green Industry, Faculty of Agro-Industry, Chiang Mai University, Chiang Mai 50100, Thailand; 3 Center of Excellence in Materials Science and Technology, Chiang Mai University, Chiang Mai 50200, Thailand; patnarin.w@cmu.ac.th; 4 Department of Rubber and Polymer Technology, Faculty of Engineering and Agro-Industry, Maejo University, Chiang Mai 50290, Thailand; siwarote.b@mju.ac.th; 5 Department of Materials Science and Technology, Faculty of Science, Prince of Songkla University, Songkhla 90110, Thailand; thorsak.k@psu.ac.th; 6 Department of Chemistry, Faculty of Science, Chiang Mai University, Chiang Mai 50200, Thailand; 7 College of Biomedical Engineering, Rangsit University, Pathum Thani 12000, Thailand; nuttapol.t@rsu.ac.th; 8 Department of Pharmaceutical Science, Faculty of Pharmacy, Chiang Mai University, Chiang Mai 50200, Thailand; warintorn.ruksiri@cmu.ac.th

**Keywords:** epoxidized natural rubber, water hyacinth fiber, chlorhexidine gluconate

## Abstract

In this study, epoxidized natural rubber (ENR) was mixed using a two-roller mixer. Water hyacinth fiber (WHF) acted as a reinforcing agent in the preparation of the rubber composite at 10 phr (ENRC/WHF). Chlorhexidine gluconate (CHG) was added at different concentrations (1, 5, 10, and 20 phr) as an antimicrobial and coupling agent. The tensile strength increased with a CHG content of 1 phr (4.59 MPa). The ENRC/WHF/CHG20 blend offered high hardness (38) and good morphology owing to the reduction in cavities and fiber pull-out from the rubber matrix. The swelling of the sample blends in oil and toluene decreased as the CHG content increased. Reactions of –NH_2_/epoxy groups and –NH_2_/–OH groups occurred during the preparation of the ENRC/WHF/CHG blend. The FTIR spectroscopy peak at 1730 cm^−1^ confirmed the reaction between the −NH_2_ groups of CHG and epoxy groups of ENR. The ENRC/WHF/CHG blend at 10 phr and 20 phr exhibited zones of inhibition against three bacterial species (*Staphylococcus aureus*, *Escherichia coli*, and *Bacillus cereus*). CHG simultaneously acted as a crosslinking agent between ENR and WHF and as an antimicrobial additive for the blends. CHG also improved the tensile strength, hardness, swelling, and antimicrobial properties of ENR composites.

## 1. Introduction

Thailand is the largest producer and exporter of natural rubber worldwide, making a major contribution to the nation’s economy. The aim of the research team was to develop and process rubber for a wide range of applications and to promote the value addition of agricultural products. In the past, many types of natural cellulose fibers have been studied as polymer-reinforcing fibers, such as coconut, jute, banana, and hemp fibers [1,2], instead of using synthetic fibers, such as glass and nanocarbon fibers [3,4], to reduce the problem of degradation and environmental pollution.

Natural rubber (NR) is a natural material used worldwide since the industrial revolution. The demand for NR has increased in many industries, especially because of its versatile applications in daily life. However, NR properties are unsuitable for practical applications. Therefore, natural rubber has been modified and processed to improve its properties for practical purposes. The mechanical properties, solvent resistance, and temperature resistance of NR can be improved via vulcanization. Sulfur, stearic acid, zinc oxide, fillers, and accelerators have been employed for the vulcanization of NR. Reinforcement fillers were used to prepare high-performance NR composites. The addition of metal filler particles to the NR matrix efficiently improves the mechanical performance, electrical properties, and chemical reactions of the NR composite. Carbon black (CB) is mainly used in the rubber industry to improve the properties of NR, whereas silica is most commonly used to reinforce NR [5]. Graphene and carbon nanotubes (CNT) have been used as fillers to improve NR properties in much research [6,7,8,9,10]. CaCO_3_, TiO_2_, and Al_2_O_3_ have also been applied to NR rubber for reinforcement it [11,12,13]. However, some concerns regarding CB and metal nanoparticles in NR production are the main issues affecting health, safety, and the environment. These compounds are produced from petroleum and can cause health problems. CB and nanometals produce greenhouse gases that are related to global warming. Therefore, CB and nanometallic particles are the main problems faced by many industries. Siliga mixed with NR is also problematic due to its hydrogen groups on the NR surface [14,15]. Natural rubber with reactive functional groups is a potential material for the development of new rubber composites because of the high reactivity of the functional groups in the structure, which react with other functional groups of natural materials. Covalent bonds and interactions between reactive functional groups can facilitate stress transfer from the matrix material by toughening the materials and improving the properties of the composite. Epoxidized natural rubber (ENR) is a renewable, soft, flexible, biocompatible, and biodegradable resource. Unlike conventional natural rubber, ENR contains an oxirane ring that exhibits high polarity [16], resulting in good resistance to various chemicals and oils. ENR is synthesized by the epoxidation reaction of natural rubber molecules to form epoxide groups at the double bond site, presenting epoxy bands at 836 and 870 cm^−1^ in FTIR spectroscopy [17,18]. ENR is a natural rubber that can be used as an elastic natural material to react with other natural materials via the ENR epoxy groups to improve its properties. Green natural materials are challenging fillers for toughening and provide some advantages to the main rubber.

Thailand is facing the problem of water hyacinth because of its rapid growth and propagation, resulting in less oxygen in bodies of water. This leads to a high risk of water pollution and difficulty in water transportation and sewage water due to the blockage of waterways. Previous studies have attempted to use water hyacinth fiber (WHF) as a reinforcing material in rubber [19,20]. This is challenging for researchers owing to the physical characteristics of WHF, which is less tough than other natural fibers, such as hemp, bamboo, and banana [2]. The use of short cellulose fibers as a filler in natural rubber compounds has been proposed to increase the tensile strength but decrease the elongation at break [21]. Therefore, to maintain the flexibility of rubber products, the fiber content should not exceed 10 phr [22]. Coupling agents are also used to improve the mechanical properties by increasing the adhesion between the rubber and fiber [21].

Chlorhexidine gluconate (CHG) is the most widely used antimicrobial agent in dentistry [23]. CHG shows limited activity against Gram-negative and Gram-positive bacteria, microbacteria, and fungi [24]. Previously, CHG was used as a cross-linking agent in reactions to improve the properties of thermoplastic starch and as an effective antimicrobial agent [18,25]. The effect of CHG on the mechanical and antimicrobial properties of ENR rubber with natural fibers as an encapsulated material is attractive for the development of new rubbers with excellent properties for a wide range of applications. The reaction between ENR and CHG is key to successful research but has not been explained in detail. High-performance ENR with mechanical, elastic, oil-resistant, and antimicrobial properties is a target for researchers to develop new active rubbers with excellent properties.

Therefore, the purpose of this study is to use WHF as a reinforcing material in ENR to develop antimicrobial natural rubber with high mechanical and elasticity properties. WHFs are also expected to absorb and release CHG, which provides antimicrobial agent to the ENR rubber matrix. CHG was selected as the crosslinking agent to enhance the reaction between the amino groups of CHG and the epoxy groups of ENR or hydroxyl groups of the fiber. The mechanical properties, elastic recovery, hardness, swelling ratio, morphology, antimicrobial activity, and reactions of ENR/WHF/CHG blends were investigated. This material is expected to apply for active cushioning packaging and medical drug carries with high tensile strength, elasticity, and antimicrobial properties.

## 2. Materials and Methods

### 2.1. Materials

ENR is a natural rubber with epoxy reactive functional groups in the structure, which contains high elasticity and chemical resistance properties. ENR with 25% epoxide (ENR–25), which was used as the matrix material, was obtained from the Muang Mai Katree Co., Ltd., Phuket, Thailand. Some crosslinking additives are used to improve properties of natural rubber by formation of crosslinking. The substances used to stabilize natural rubber, including zinc oxide, were purchased from Suksapanpanit, Bangkok, Thailand. Stearic acid used as the activator was purchased from the Union Science Co., Ltd. (Chiang Mai, Thailand). N–cyclohexyl–2–benzothiazole sulfenamide (CBS), a delayed-action accelerator that produces rubber vulcanizates with a high modulus and high tensile strength, and tetramethylthiuram disulfide (TMTD), an ultra-accelerator for low-temperature curing, were obtained from the Lucky Four Co., Ltd., Bangkok, Thailand. Sulphur was purchased from the SCITRADER Co., Ltd. (Bangkok, Thailand). Water hyacinth fiber is used to develop natural fiber products due to high cellulose and a minimum of hemicellulose content. Water hyacinths were obtained from Irrigation Water Management Experiment Station 1 (Padad), Chiang Mai, Thailand. CHG is an antiseptic additive, which is used in skin disinfection due to its antimicrobial properties. CHG was purchased from the S. Tong Chemical Co., Ltd. (Chiang Mai, Thailand).

### 2.2. Preparation of Water Hyacinth Fibers

The washed water hyacinth stems were cut into small pieces of 5 × 5 cm, sun dried for 2 days, and dried at 105 ± 3 °C for 3 h to remove moisture. The dried water hyacinths were shredded lengthwise, and the pulp was boiled with 12% (*w*/*v*) sodium hydroxide at a 1:20 ratio at 85 ± 5 °C for 3 h. The resulting slurry was vacuum filtered and rinsed with distilled water until the pH reached 6.5–7. Fibers were dried at 70 ± 3 °C for 3 h, while lignin and hemicellulose were removed by bleaching the fiber (200 g) with a 10% (*v*/*v*) hydrogen peroxide solution at to 85 ± 5 °C for 3 h. The bleached fiber was washed to achieve a neutral pH (6.5–7) and dried at 70 ± 3 °C for 2 h. Bleaching was repeated twice, and the fibers were ground to a 250 μm size, followed by storage in a desiccator. 

### 2.3. Sample Preparation

ENR, crosslinking agents, WHF, and CHG were melt-blended using a two-roll mill (Pirom–Olarn Co. Ltd., Bangkok, Thailand, PI–140) at room temperature with a speed of 50 rpm for 25 min to prepare ENR, ENRC, and ENRC/WHF/CHG blends with CHG mixing ratios of 1, 5, 10, and 20 phr. The samples were compressed into sheets using a hot compress at 130 °C at 3000 psi for 10 min and used for further analysis. The codes and compositions of the samples are listed in Table 1. 

### 2.4. Reaction Mechanism

A Fourier transform infrared spectrometer (FT/IR–4700, Jasco Corp., Tokyo, Japan) was used to test the reactions of the functional groups in ENRC/WHF/CHG by investigating thin films of the samples in the spectral range of 500–4000 cm^−1^.

### 2.5. Tensile Properties

The tensile properties were observed according to the JIS K 6251–7 standard using a tensile tester (Model H1KS, Hounfield Test Equipment, Surrey, England) at a crosshead speed of 50 mm/min. The shaped samples were prepared as sheets by compression molding at 130 °C for 10 min. The dimensions of the samples were 5 mm × 40 mm × 1 mm (width × length × thickness).

### 2.6. Elastic Recovery

The elastic recovery was measured following the JIS K 6251–7 standard using a tensile tester (Model H1KS, Hounfield Test Equipment, Surrey, England). The sample was pulled up to 100% and returned to its original position to obtain the stress-strain curves. A gap length of 20 mm and test speed of 50 mm/min were used. The dimensions of the samples were 5 mm × 40 mm × 1 mm (width × length × thickness).

### 2.7. Shore A Hardness Test

The Shore A hardness was measured to evaluate the hardness of the samples at 15 s using ASTM D2240 with a specimen thickness of 6 mm. The hardness was measured at five positions on each sample at room temperature using a Shore durometer hardness tester (E2-D; Imada Co., Ltd., Toyohashi, Japan).

### 2.8. Swelling Ratio

Swelling tests were performed according to the ASTM D3616 standard. The swelling ratio was measured by cutting the samples to dimensions of 10 mm × 10 mm × 1 mm (width × length × thickness). The samples were immersed in toluene and palm oil for 48 h and then weighed (Ws) using an electronic balance. The swelling ratio was calculated using the following equation:

(1) $$\mathrm{Swelling}\;\mathrm{ratio}=\frac{W_{b}-W_{a}}{W_{a}}\;\times 100$$

where W_a_ and W_b_ are the weights of the unswollen and swollen rubber, respectively.

### 2.9. Morphology

The morphologies of the blended samples were characterized by scanning electron microscopy (SEM; JSM–5910LV JEOL Co., Ltd., Tokyo, Japan) at 15 kV. The samples were broken in liquid nitrogen, and the fracture surface was coated with a thin layer of gold by sputtering (108 Auto/SE sputter coater, Cressington Co., Ltd., Watford, England). The sample dimensions were 5 mm × 40 mm × 1 mm (width × length × thickness).

### 2.10. Antimicrobial Activity

The antimicrobial efficacies of the samples were tested against three bacterial species (*S. aureus*, *E. coli*, and *B. cereus*) and three fungal species (*A. oryzae*, *R. oligosporus*, and *S. cerevisiae*) using agar disc diffusion. Penicillin and ketoconazole were used as reference standards (positive controls) for bacteria and fungi, respectively. For the agar disc diffusion method, test samples were cut into 3 mm diameter discs, while positive control (10 µL) was applied on 3 mm sterile filter discs. Plain filter discs (negative controls) were placed on agar plates that were uniformly pre-swabbed with microbial suspensions (equivalent to approximately 10^7^ CFU mL^−1^ from 24 h grown cultures). The agar plates were incubated for 24 h at 37 °C for microbial growth. The diameter of the inhibition zone was measured to determine antimicrobial activity. Three replicate samples were analyzed for each microbe.

### 2.11. Statistical Analysis

One-way ANOVA using the Statistical Package for the Social Sciences was used to analyze the data. Differences (*p* < 0.05) were evaluated using the Duncan test.

## 3. Results and Discussion

### 3.1. Reaction Mechanism

**Figure 1:**
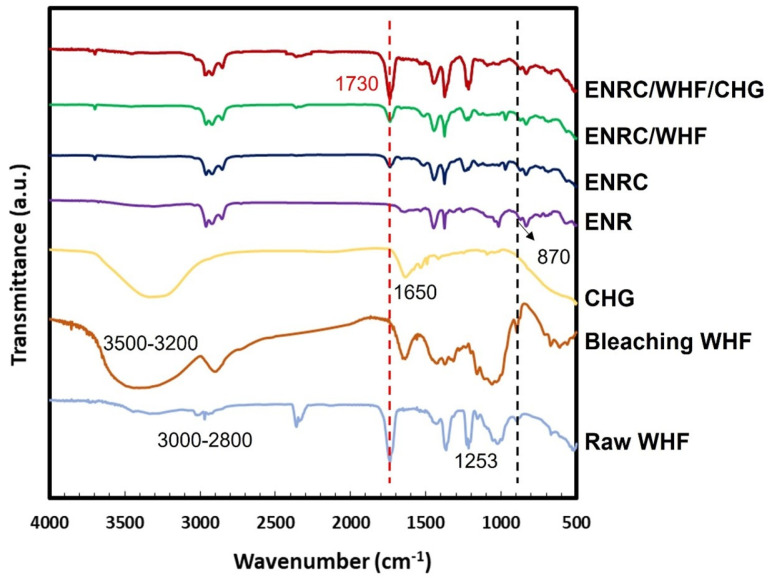
FTIR spectra of raw WHF, bleached WHF, CHG, ENR, ENRC, ENRC blended with WHF and ENRC/WHF blended with CHG.

**Figure 2:**
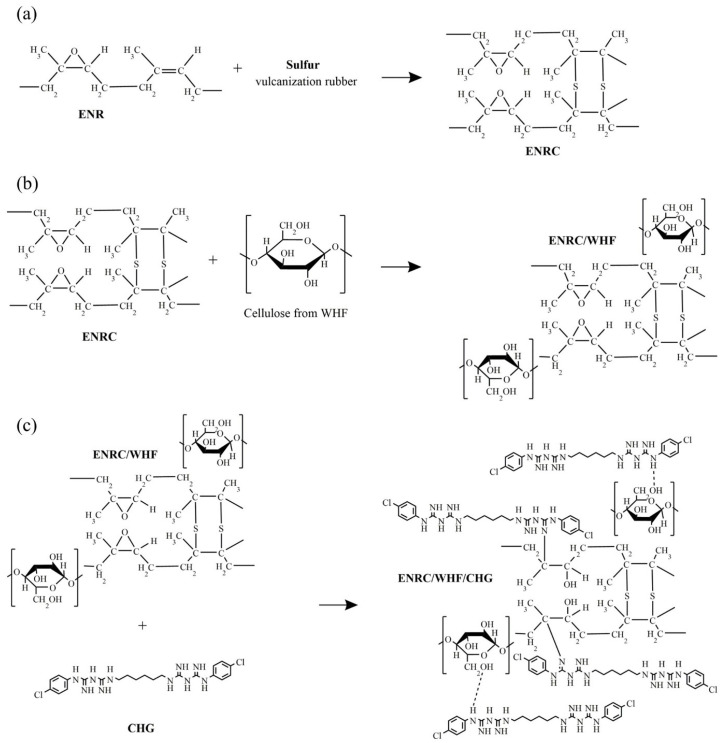
The proposed reaction of (**a**) ENR and sulfur in a vulcanization process, (**b**) ENRC and WHF, and (**c**) ENRC/WHF/CHG.

The FTIR spectra of raw WHF, bleached WHF, CHG, ENR, ENRC, and ENRC blended with WHF and CHG are shown in Figure 1. The raw fibers and fibers treated with alkaline solution showed a stretching vibration of the hydroxyl group O−H at 3200–3500 cm^−1^. In addition, the C–H groups showed peaks at 2800 and 3000 cm^−1^, which indicates the presence of cellulose molecules [26]. The constituents of lignin present in the raw fibers showed peaks at 1253, 1534, 1697, 1600, 1510, and 837 cm^−1^; however, the only peak that could be used to estimate lignin removal was at 1510 cm^−1^. The lignin peak of the alkali-treated WHF, which corresponds to the vibrations of the aromatic skeletal and carbonyl groups [19], disappeared, indicating that most of the lignin was removed after the alkaline treatment [27,28]. After treatment with alkali and bleaching, these three peaks decreased [29,30]. ENR showed symmetric and asymmetric epoxide bands, with C=CH wagging at 836 cm^−1^ and C–O–C stretching of the partial ring opening of the epoxide group at 870 cm^−1^ [31]. The bands at 1377 and 1448 cm^−1^ were attributed to stretching of the −CH_3_ and C–H groups, whereas bands at 2854, 2917, and 2961 cm^−1^ were due to C–H stretching and −CH_2_ groups, respectively [17,18,31,32]. CHG showed the characteristic peaks of N–H stretching at 1580 cm^−1^ and C–N stretching at 1650 cm^−1^. The peaks at 1095 cm^−1^ and 1155 cm^−1^ were attributed to the stretching of chloride bonded to the aromatic ring and C–O–C, respectively [18,25]. The reduction of lignin allowed the fibers to crosslink with ENR via hydrogen bonding between the hydroxyl groups and the epoxy ring groups of ENR. ENRC presented two new peaks at 1650 and 980 cm^−1^ due to the crosslinking reaction of ENR from rubber crosslinking agent. The ENRC/WHF spectra were similar to those of ENRC. The addition of CHG to ENRC/WHF resulted in a reduction of the 980 cm^−1^ peak and an increase in peak intensity at 1730 cm^−1^ owing to the occurred reactions. The reaction between the amine and epoxy groups in ENR produced a strong covalent bond crosslinks, which presented a new C–O overlap peak vibration at 1730 cm^−1^ [18,33]. Figure 2 shows the proposed reactions for the blends. These reactions improved the properties of the ENR composites.

### 3.2. Tensile Properties

**Figure 3:**
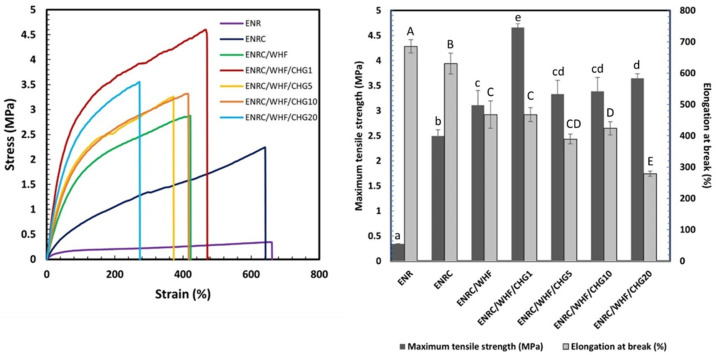
Stress-strain curves of ENR, ENRC, ENRC/WHF and ENRC/WHF blended with 1, 5, 10, and 20 phr of CHG; *n* = 10. Different lowercase superscript letters and uppercase letters indicate significant difference (*p* < 0.05) of maximum tensile strength and elongation at break, respectively.

The mechanical properties of the ENR composites in the presence of WHF and CHG at 0, 1, 5, 10, and 20 phr are shown in Figure 3. ENR exhibited a low tensile strength (0.3 MPa) and high elongation at break (700%). ENRC presented an increase in the tensile strength to 2.5 MPa with an elongation at break of 650%, owing to the occurrence of crosslinking resulting from the addition of crosslinking agents to the ENR rubber. ENRC/WHF exhibited a tensile strength of 3 MPa and an elongation at break of 460%. The reinforcement of the ENRC matrix with WHF at 10 phr resulted in high tensile strength owing to the connection of the fibers obtained from the alkaline treatment and bleaching process with the ENR matrix and absorbed strength. This was due to the roughness and interaction of the fiber surface with the ENR rubber, which increased the bonding force between the WHF and ENRC. ENRC/WHF with 1 phr CHG showed a maximum tensile strength of 4.6 MPa and an elongation at break of 471%, which resulted in a higher tensile strength than ENR with and without CHG. The structure of the samples was improved, and they presented the ability to transfer stress at the interface between the WHF and matrix. In addition, 10 phr CHG was sufficient to enhance the interfacial adhesion between the WHF and ENRC [29,34] and improve its tensile properties. A high amount of CHG reduced the tensile strength because of the excessive amount of CHG, which acted as a defect in the composite. The combination reactions of crosslinking inside ENRC via a sulfur crosslinking agent and the reaction between the amino (–NH_2_) groups of CHG and epoxy groups of ENR improved the mechanical properties of ENR composites.

### 3.3. Elastic Recovery

**Figure 4:**
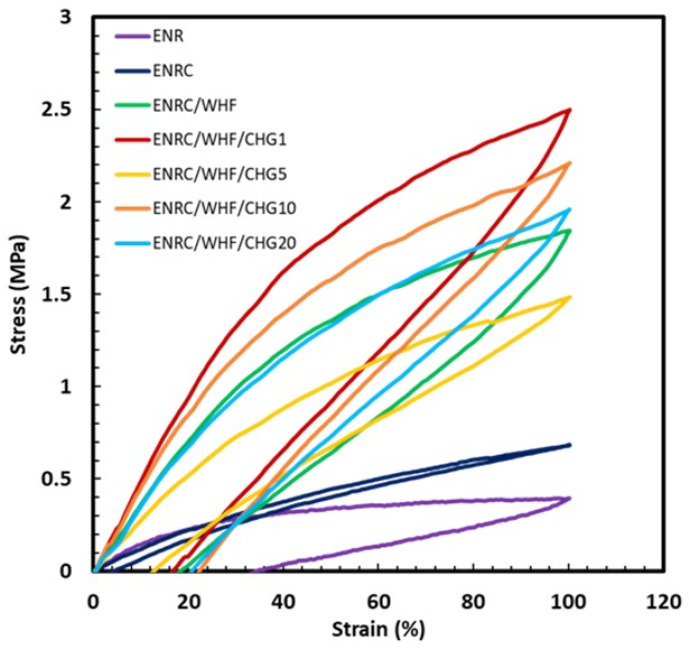
The elastic recovery of ENR, ENRC, ENRC/WHF, and ENRC/WHF blended with 1, 5, 10, and 20 phr of CHG.

The elastic recovery of the rubber composite was tested by stretching it to 100% and then returning it to its starting position at a constant speed. The results of the elastic recovery are shown in Figure 4. ENR, ENRC with crosslinking agent, and ENRC/WHF showed strain recoveries of 30, 5, and 20%, respectively. The ENRC/WHF samples containing CHG at 1 and 5 phr showed strain recoveries of 13–14%, whereas CHG at 10 and 20 phr presented a strain recovery of 20–25%. The improvement in strain recovery for the ENRC/WHF/CHG samples indicated enhanced interfacial adhesion and compatibility between the rubber and fiber via a reaction induced by CHG. The enhanced elastic recovery of the blends is due to the reaction between the –NH_2_ groups of CHG and epoxy groups of ENR [18]. A suitable amount of CHG (1–5 phr) was used to induce the optimal reactions inside the polymer composite. Excessive amounts of CHG (10–20 phr) reduced the elastic recovery compared to the addition of CHG 1–5% due to its incompatibility effect. The ENRC/WHF/CHG5 showed the highest elastic recovery with reduction of tensile strength (Figure 3) due to combination effect between high rubber crosslinking density and excessive amount of CHG.

### 3.4. Shore A Hardness

**Figure 5:**
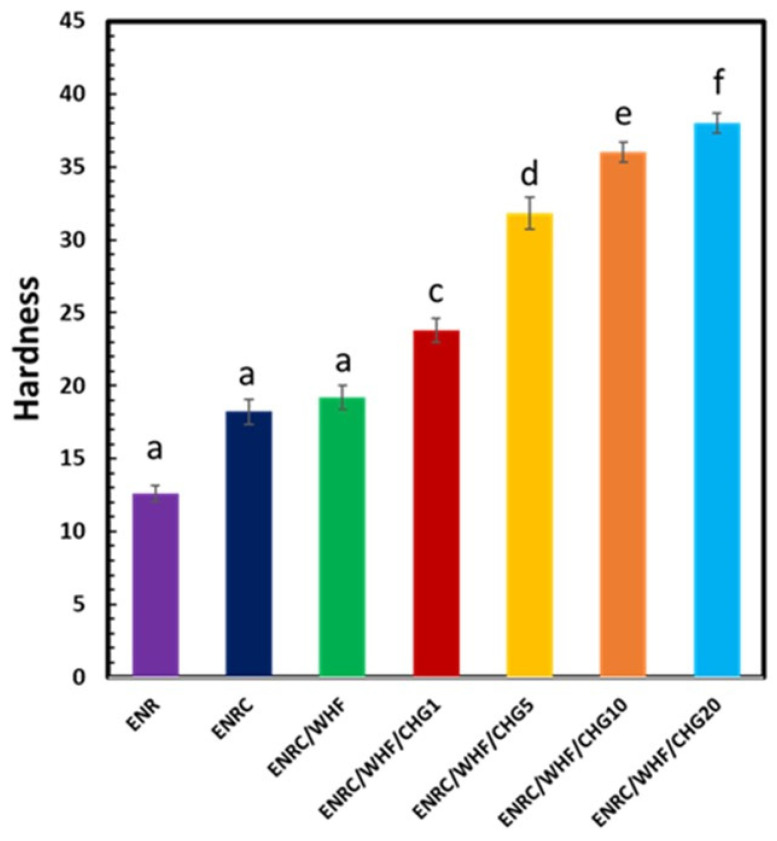
Shore A hardness of the ENR, ENRC, ENRC/WHF and ENRC/WHF blended with 1, 5, 10 and 20 phr of CHG; *n* = 5. Different lowercase superscript letters indicate significant difference (*p* < 0.05) of hardness.

The hardness values of the samples obtained from the durometer test are shown in Figure 5. The Shore A hardness of ENR was 12.6, while those of ENRC, ENRC/WHF, and ENRC/WHF blended with CHG at 1, 5, 10, and 20 phr were 18.2, 19.2, 23.8, 31.8, 36, and 38, respectively. Hardness increased with increasing CHG content. This was due to the interaction of hydrogen in the fiber, the crosslinking between the amino groups (–NH_2_) of CHG, and the reaction between the epoxy groups of ENR and the amino groups (–NH_2_) of CHG. This can be explained by the homogeneous dispersion in these samples observed in the SEM images and the improvement in the CHG and ENR reactions [18]. These reactions indicated a high crosslinking density inside the rubber, which increased the density and hardness of the composite.

### 3.5. Swelling Ratio

**Figure 6:**
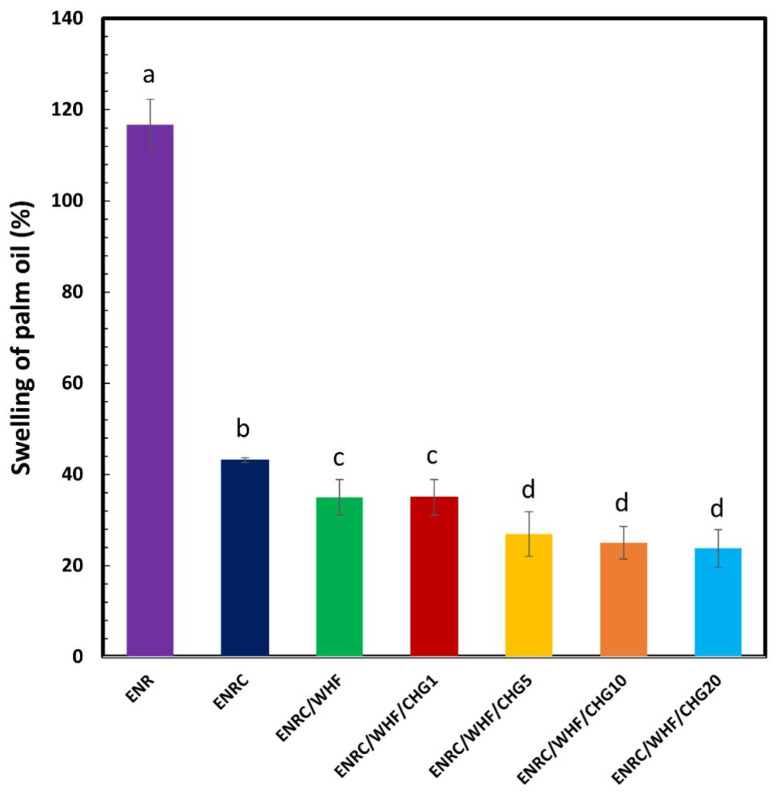
Swelling ratio of ENR, ENRC, ENRC/WHF and ENRC/WHF blended with 1, 5, 10 and 20 phr of CHG; *n* = 5. Different lowercase superscript letters indicate significant difference (*p* < 0.05) of swelling in palm oil.

The crosslinking efficiency of the rubber composite samples was assessed using the swelling ratio, which is an indirect measure of the cross-linking-related density of the material. Figure 6 shows the swelling ratios of ENR, ENRC, ENRC/WHF, and ENRC/WHF with different concentrations of CHG (1, 5, 10, and 20 phr) in palm oil. The swelling of the rubber indicates the permeability of palm oil. The swelling ratios of ENR, ENRC, ENRC/WHF, and ENRC/WHF with CHG at 1, 5, 10, and 20 phr in palm oil were 116.7, 43.2, 35.0, 35.0, 26.9, 25.0, and 23.8%, respectively. The swelling properties showed a low oil resistance with a high swelling ratio of ENR owing to the low polarity of ENR and easy absorption of ENR in nonpolar oil [31,35]. However, swelling decreased when crosslinking agents were added to ENR and decreased with increasing CHG content owing to the improvement in crosslinking within the dense molecular chains of rubber. The diffusion rate of palm oil into rubber was also reduced with a high density of crosslinked samples. Some studies have also demonstrated that an increase in the degree of crosslinking density provides a low degree of swelling of rubber [18,31]. A low movement of the crosslinked ENR rubber was observed, which provided a low degree of swelling of the samples. Combination crosslinking in ENRC from the C=C of rubber with sulfur and –NH_2_ groups of CHG with epoxy groups of ENR were synergistic reactions that reduced swelling and improved the oil resistance of ENR rubber. The low movement of the ENR chain with a high crosslinking network via both types of crosslinking provided oil-resistant properties to the ENR composites.

### 3.6. Morphology

**Figure 7:**
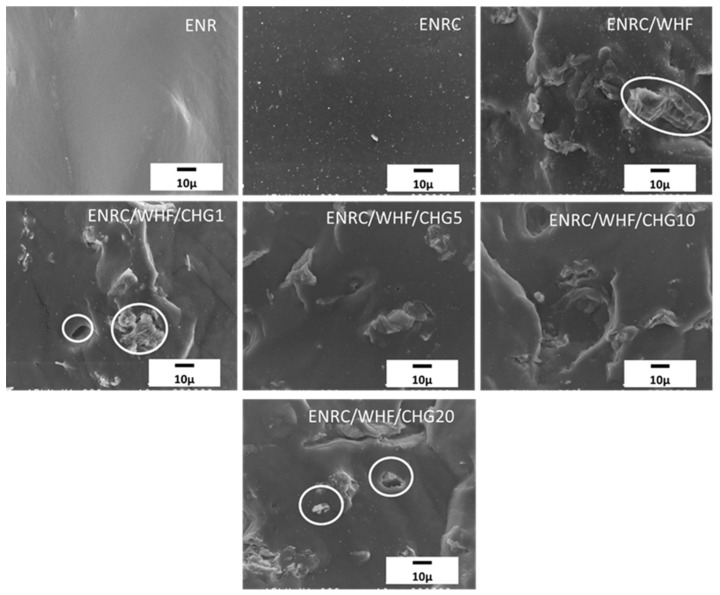
SEM fracture surface images of ENR, ENRC, ENRC/WHF and ENRC/WHF blended with 1, 5, 10 and 20 phr of CHG.

The morphologies of the rubber composites with CHG contents of 1, 5, 10, and 20 phr were studied using SEM. The fracture surface morphologies of the samples are shown in Figure 7. Granular particles were observed throughout the rubber samples with crosslinking agent. In the ENRC/WHF with fiber filling, numerous cavities and pulled-out fibers were observed, which are characteristic of ENR. The presence of these cavities and pulled-out fibers confirmed that the interfacial bonding between the filler and rubber matrix was poor and weak [29,33]. ENRC/WHF/CHG showed a smooth rubber phase surface with good interfacial adhesion between the fibers and the matrix. This was due to the strong interaction within the polymer chain between the hydroxyl groups of the fibers and amine groups of CHG and the reaction between the amine groups of CHG and epoxy groups of ENR [17,18,25,33]. These results indicate improved mechanical properties owing to the strong fiber interfacial adhesion with the ENR rubber, which improved the mechanical properties and elasticity of the ENR composites. In the ENRC/WHF/CHG1 sample, suitable reaction between epoxy groups of ENR and –NH groups of CHG induced crosslinking network of ENR, while strong interaction between –OH groups of WHF with –NH groups of CHG provided high interfacial adhesion between ENR rubber and WHF surface. This reaction mechanism provided the SEM image of smooth fracture surface with strong interfacial adhesion between rubber and fiber, which presented highest tensile (Figure 3) and excellent elastic recovery (Figure 4).

### 3.7. Antimicrobial Activity

**Figure 8:**
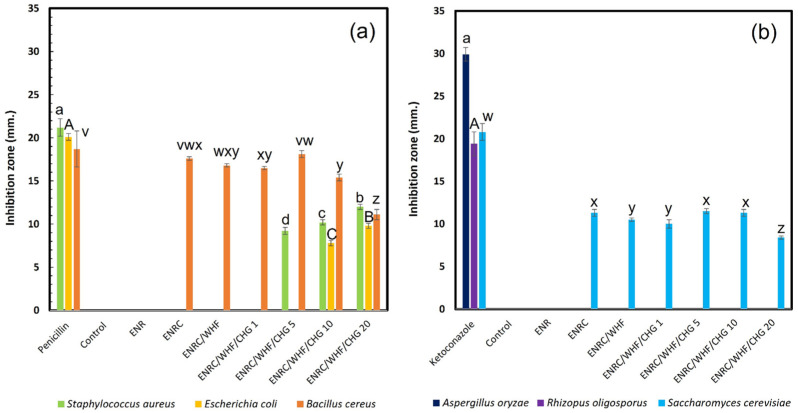
Inhibition zones of ENR, ENRC, ENRC/WHF and ENRC/WHF blends with CHG at 1, 5, 10 and 20 phr: (**a**) bacteria and (**b**) fungi; *n* = 3. Different A–C uppercase letters (*E. coli* and *R. oligosporus*), a–d lowercase letters (*S. aureus* and *A. oryzae*), and v–z lowercase letters (*B. cereus* and *S. cerevisiae*) indicate significant difference (*p* < 0.05) of inhibition zone.

**Figure 9:**
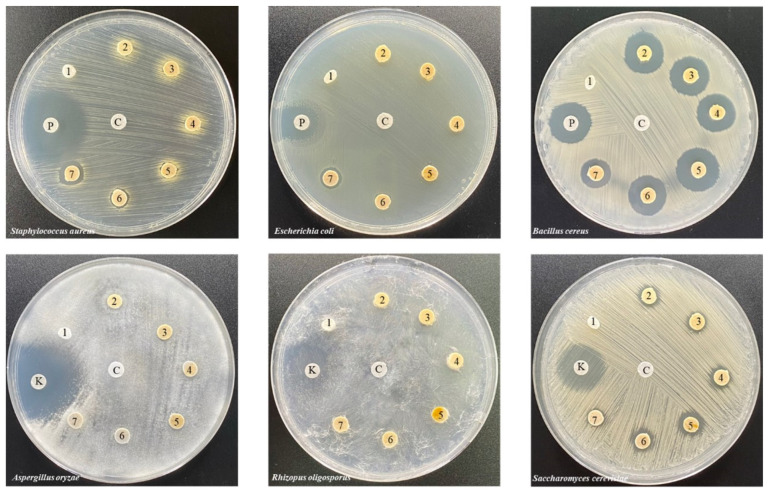
Images of the inhibition zones of ENR (1), ENRC (2), ENRC/WHF (3), ENRC/WHF blends with CHG at (4) 1 phr, (5) 5 phr, (6) 10 phr, (7) 20 phr, (P) positive control with penicillin, (K) positive control with ketoconazole, and (C) negative control on the microbial activity of bacteria (*S. aureus*, *E. coli*, and *B. cereus*) and fungi (*A. oryzae*, *R. oligosporus*, and *S. cerevisiae*).

Antimicrobial activity was assessed by examining the zone of inhibition in the growth area. The antimicrobial activity results are shown in Figure 8 and Figure 9. The ENR sample was found to have no inhibition zone against the microbial species tested, whereas the ENRC and ENRC/WHF samples inhibited both (*B. cereus*) and (*S. cerevisiae*) because zinc oxide (ZnO), which is used as a stimulant in vulcanized rubber, possesses antimicrobial properties. The ENRC/WHF/CHG1 blend exhibited inhibition zones against both (*B. cereus*) and (*S. cerevisiae*). The ENRC/WHF/CHG5 sample inhibited the growth of the bacteria *S. aureus* and *B. cereus* and fungal species *S. cerevisiae*. The ENRC/WHF/CHG 10 and 20 phr samples showed inhibition zones against all bacteria and the fungal species *S. cerevisiae*. The inhibitory performance of the composite rubber was affected by the addition of ZnO and CHG, which have antimicrobial activity [17,25,35]. The high antimicrobial properties of ENR/WHF/CHG 10 and 20 phr samples were due to the high CHG content absorbed by WHF. WHF acted as an encapsulation material for antimicrobial agents, which enabled the release of antimicrobial agents to the bacteria. The antifungal effect of ENRC was demonstrated by the ZnO catalyst acting as a cross-linking agent against *S. cerevisiae*. ZnO inhibition of *S. cerevisiae* has been reported [36,37]. The inhibition zone of the fungal test was observed due to the diffusion of CHG with the interacting ZnO. The mechanical properties and elasticity of ENRC/WHF/CHG samples were improved via the CHG and ENRC crosslinking reactions, while the excess CHG absorbed in WHF provided good antimicrobial properties for the ENR composites.

## 4. Conclusions

ENR blends with WHF and CHG were successfully developed with improved mechanical, oil swelling, and antimicrobial properties. The tensile strength of ENRC/WHF/CHG increased with CHG at 1 phr owing to the optimal crosslinking inside the ENR rubber. However, the tensile strength and elongation at break decreased with increasing CHG content owing to the excessive amount of CHG. The elastic recovery test of the composite rubber showed high strain recovery when CHG was added to the ENRC/WHF samples because of crosslinking. The interfacial adhesion between ENR and WHF was improved by alkalization of the fibers. The FTIR results confirmed that the lignin in the raw fiber was eliminated by the fiber treatment process, ENRC crosslinking occurred from the rubber crosslinking agents, and the amine groups of CHG reacted with the epoxy groups of ENR and interacted with the hydroxyl groups of cellulose. The morphology of ENRC/WHF with increased CHG content showed fewer cavities and pulled-out fibers from the ENR matrix. The addition of CHG at 10 and 20 phr inhibited all the bacterial and the fungal species *S. cerevisiae*. WHF acted as an encapsulation material for antimicrobial agents, providing releasing properties to the microbial growth agent. ENRC/WHF/CHG blends with good mechanical and antimicrobial properties will be accomplished for a wide range of packaging, agricultural, and medical industrial applications. Improvement of mechanical properties with high antimicrobial properties from double chemical crosslinking can apply to improve properties of other natural polymers blend and composite, while double effect of CHG from high reactivity functional groups and its antimicrobial properties are able to use for developing bioactive film, hydrogel, and electro spinning fiber.

## Figures and Tables

**Table 1:** Code names and compositions of ENR, ENRC, ENRC/WHF, and CHG blends (phr).

Sample Code	ENR	ENRC	ENRC/ WHF	ENRC/ WHF/ CHG1	ENRC/ WHF/ CHG5	ENRC/ WHF/ CHG10	ENRC/ WHF/ CHG20
ENR	100	100	100	100	100	100	100
ZnO	-	5	5	5	5	5	5
Stearic acid	-	1	1	1	1	1	1
CBS	-	1	1	1	1	1	1
TMTD	-	0.5	0.5	0.5	0.5	0.5	0.5
Fiber	-	-	10	10	10	10	10
CHG	-	-	-	1	5	10	20
Sulfur	-	2	2	2	2	2	2

phr unit: part per hundred of rubber.

## Data Availability

The data presented in this study are available upon request from the corresponding author.

## Abbreviations and Symbols

ENR, Epoxidized natural rubber; CHG, Chlorhexidine gluconate; WHF, Water hyacinth fibers; ENRC, Epoxidized natural rubber blend with additives; TMTD, Tetramethyl thiuram disulfide; FTIR, Fourier-transform infrared spectroscopy; SEM, scanning electron microscopy.
